# Supplementary material for: Observation of Kekulé vortices around hydrogen adatoms in graphene
Source: Nat Commun. 2024 Apr 4;15:2927. doi: 10.1038/s41467-024-47267-8 (PMC10995122; doi:10.1038/s41467-024-47267-8)
Supplement: Supplementary file 1 — Supplementary Information [file 41467_2024_47267_MOESM1_ESM.pdf]

Supplementary Information:
Observation of Kekulé vortices induced by hydrogen adatoms in graphene

Yifei Guan,[1] Clément Dutreix,[2] Hector González-Herrero,[3,4] Miguel M. Ugeda,[5,6,7] Ivan Brihuega,[3,4,8,*] Mikhail I. Katsnelson,[9] Oleg V. Yazyev,[1,†] and Vincent T. Renard[10,‡]

[1]*Institute of Physics, École Polytechnique Fédérale de Lausanne (EPFL), CH-1015 Lausanne, Switzerland*
[2]*Université de Bordeaux, France and CNRS, LOMA, UMR 5798, Talence, F-33400, France*
[3]*Departamento de Física de la Materia Condensada, Universidad Autónoma de Madrid, E-28049 Madrid, Spain*
[4]*Condensed Matter Physics Center (IFIMAC), Universidad Autónoma de Madrid, E-28049 Madrid, Spain*
[5]*Donostia International Physics Center (DIPC), Paseo Manuel de Lardizábal 4, 20018 San Sebastián, Spain*
[6]*Centro de Física de Materiales (CSIC-UPV-EHU), Paseo Manuel de Lardizábal 5, 20018 San Sebastián, Spain*
[7]*Ikerbasque, Basque Foundation for Science, 48013 Bilbao, Spain*
[8]*Instituto Nicolás Cabrera, Universidad Autónoma de Madrid, E-28049 Madrid, Spain*
[9]*Radboud University, Institute for Molecules and Materials, Nijmegen, The Netherlands*
[10]*Univ. Grenoble Alpes, CEA, Grenoble INP, IRIG, PHELIQS, 38000 Grenoble, France*

This document contains Supplementary Information for the article "Observation of Kekulé vortices induced by hydrogen adatoms in graphene". The document starts with the supplementary figures mentioned in the main text. It also contains the derivation of Eq. 1 of the main text, additional theoretical considerations and finally a description of the method used to extract the Kekulé vortex signal from the STM images.

Supplementary figures

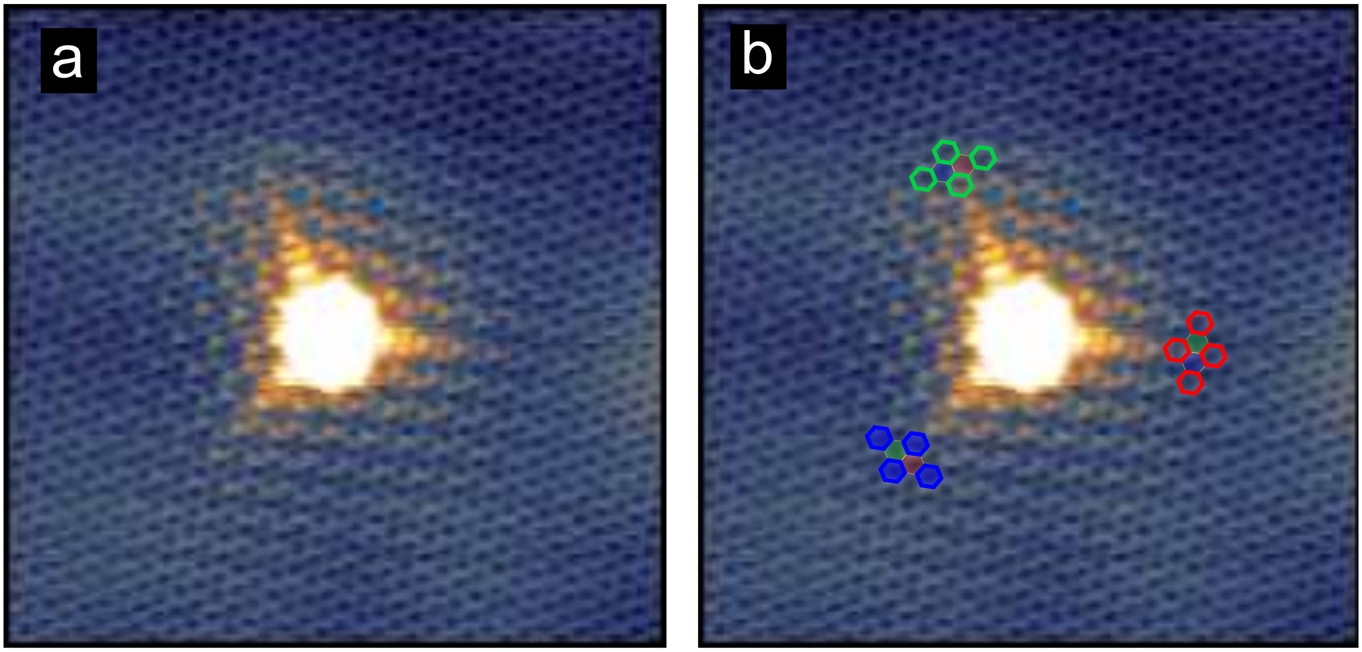

Supplementary Figure 1: **Kekulé vortex around another H atom. a,** STM image of another H atom measured in a different experimental run. The tunneling conditions and image size are the same as in Fig. 1 of the main text. **b,** Same image with the rgb tiling.

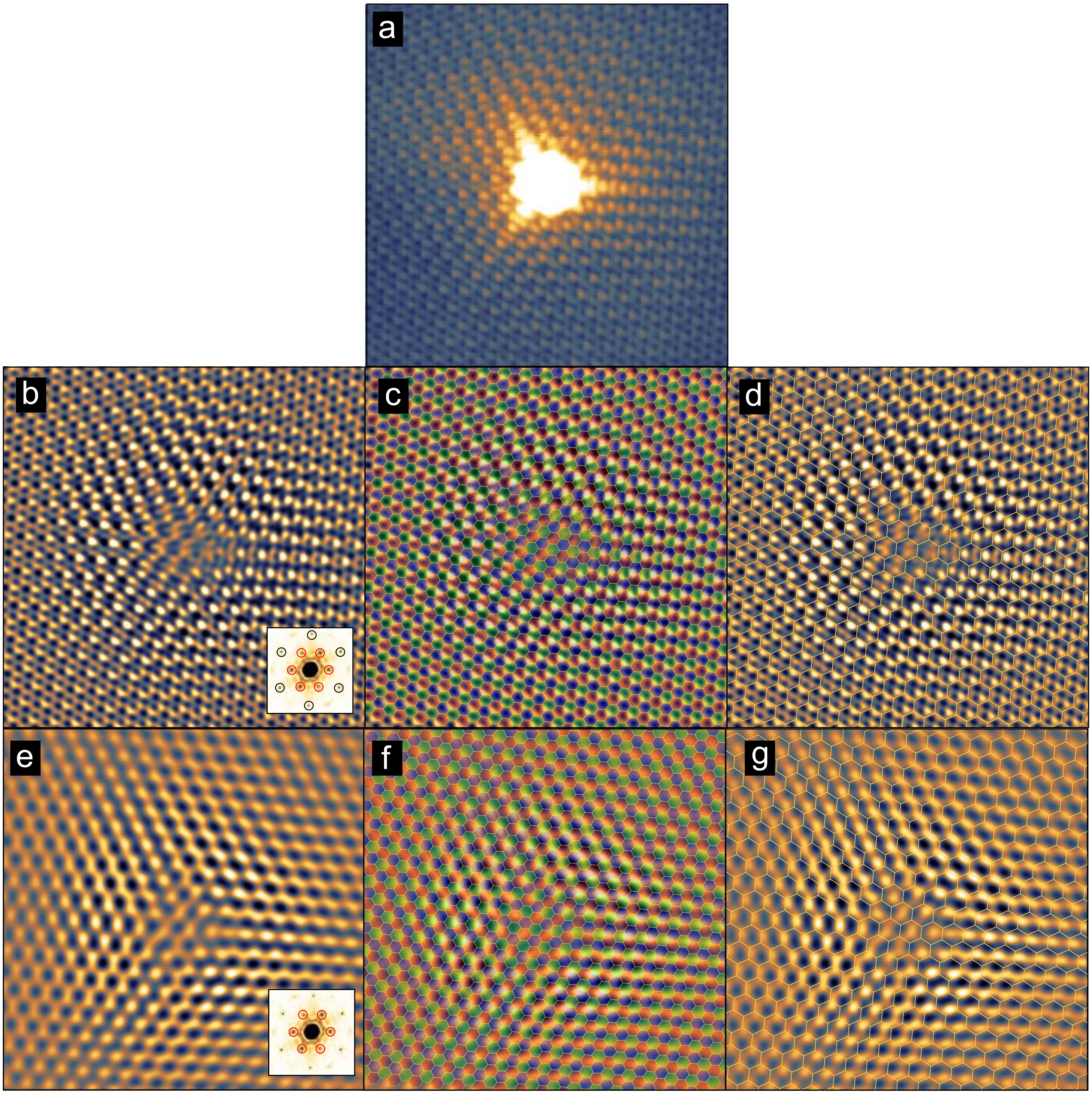

Supplementary Figure 2: **Kekulé paving of the STM image**. **a,** Raw STM image of the H atom on graphene from which Fig. 1c of the main text is obtained. The Kekulé bond order is seen far from the bright protrusion (the H atom) in the center. The image is 7.4×7.4 nm^2 in size and was measured with V_b=400 mV and i_t = 45.5 pA. **b,** Fourier filtered image including the intervalley scattering and graphene signal (see harmonics selected in the inset). **c,** Filtered image with a red, blue, green paving of graphene lattice. **d,** Filtered image with a $\sqrt{3} \times \sqrt{3}R30^\circ$ lattice superimposed. **e,** Fourier filtered image including the intervalley scattering only (see harmonics selected in the inset). **f,** Filtered image with a red, blue, green paving. **g,** Filtered image with a $\sqrt{3} \times \sqrt{3}R30^\circ$ lattice superimposed.

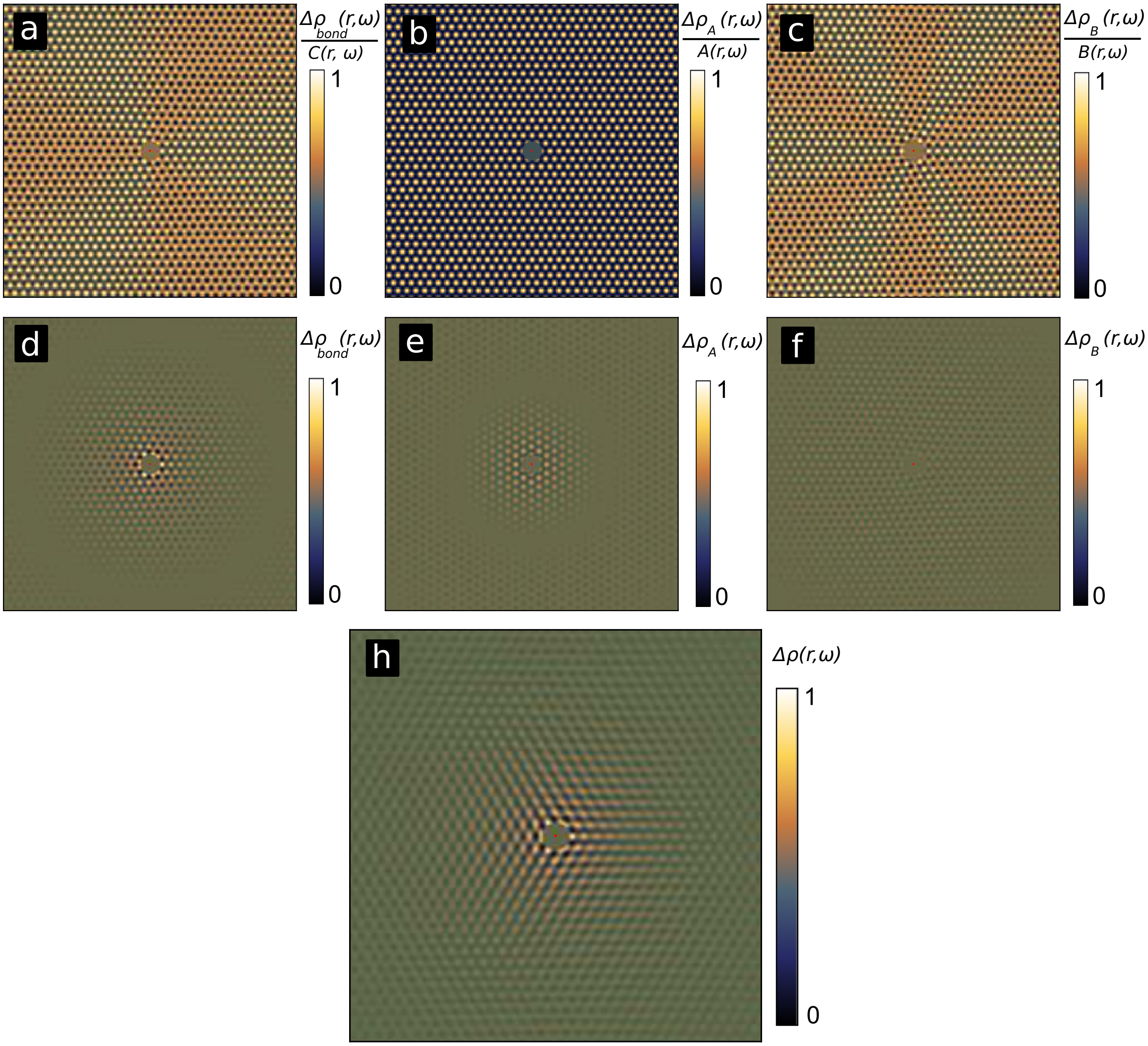

Supplementary Figure 3: **Different contributions to the LDOS calculated at 150 meV. a,** Image of normalized $\Delta\rho_{bond}(r,\omega) = \Delta\rho_{AB}(r,\omega) + \Delta\rho_{BA}(r,\omega)$. The signal contains a 2π vortex. **b,** Image of normalized $\Delta\rho_A(r,\omega)$. The signal does not contains a vortex. **c,** Image of normalized $\Delta\rho_B(r,\omega)$. The signal contains a 2π vortex. **d,** Image of $\Delta\rho_{bond}(r,\omega)$. **e,** Image of normalized $\Delta\rho_A(r,\omega)$. **f,** Image of normalized $\Delta\rho_B(r,\omega)$. **h** Sum of the contributions shown in panels **d, e** and **f**. The signal is integrated between 0 and qV_{bias} to reproduce the conditions of the STM images in the main text. The colormap in **d, e** and **f** corresponds to that of **h,** to highlight the weight of each contribution in the total signal. See the Supplementary Information document for the derivation of each contribution. The image areas are 14×14 nm^2.

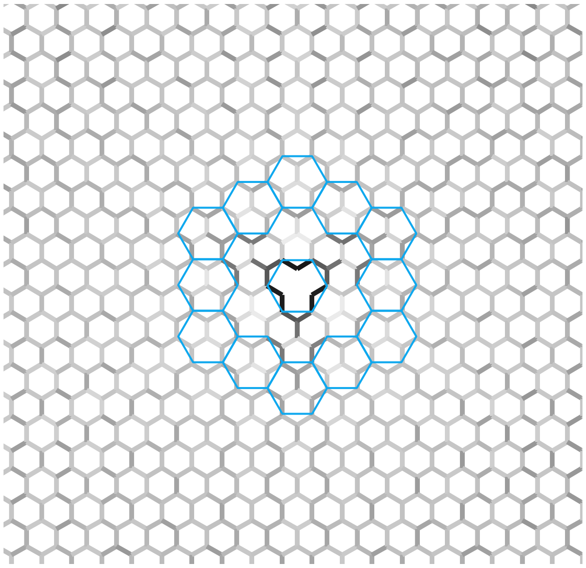

Supplementary Figure 4: **Bond order at the hydrogen adatom from tight-binding calculations.** The hexagons covering $\sqrt{3} \times \sqrt{3}$ Wigner-Seitz cells serve as a guide-of-eyes to track the evolution of the Kekulé order.

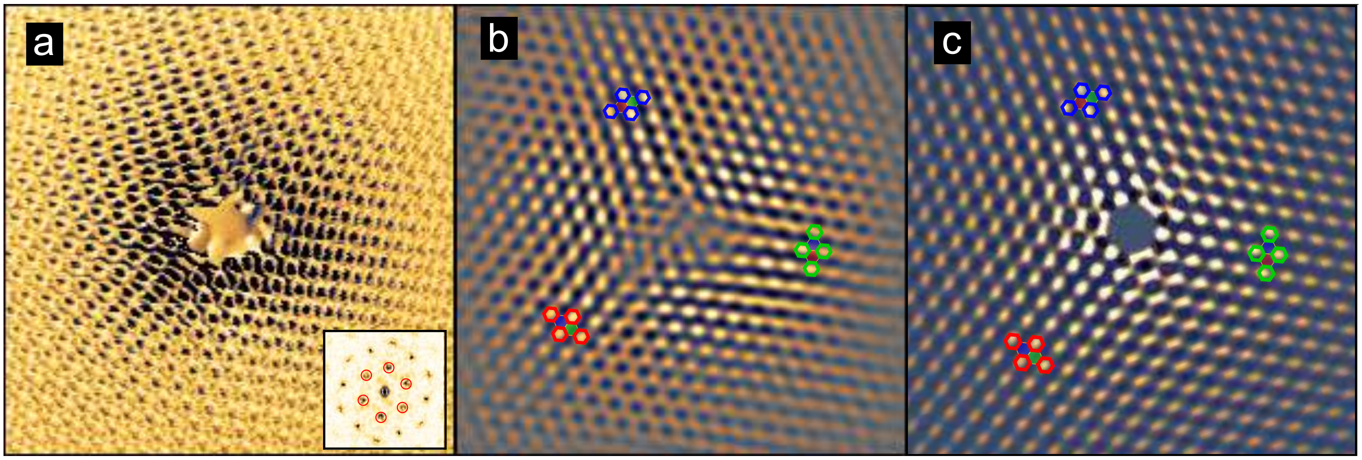

Supplementary Figure 5: **Energy resolved images of a hydrogene adatom on graphene. a,** Energy resolved STM image measured by phase sensitive detection with a 2 mV rms ac voltage at 830 Hz. The tunneling parameters are $i_t = 80$ pA and $V_b = 50$ mV. The inset shows the Fourier filter used to produce panel **b**. **b,** Fourier filtered image allowing to discriminate the three Kekulé orders. **c,** Total theoretical LDOS calculated at 50 meV as described in the Supplementary Information.

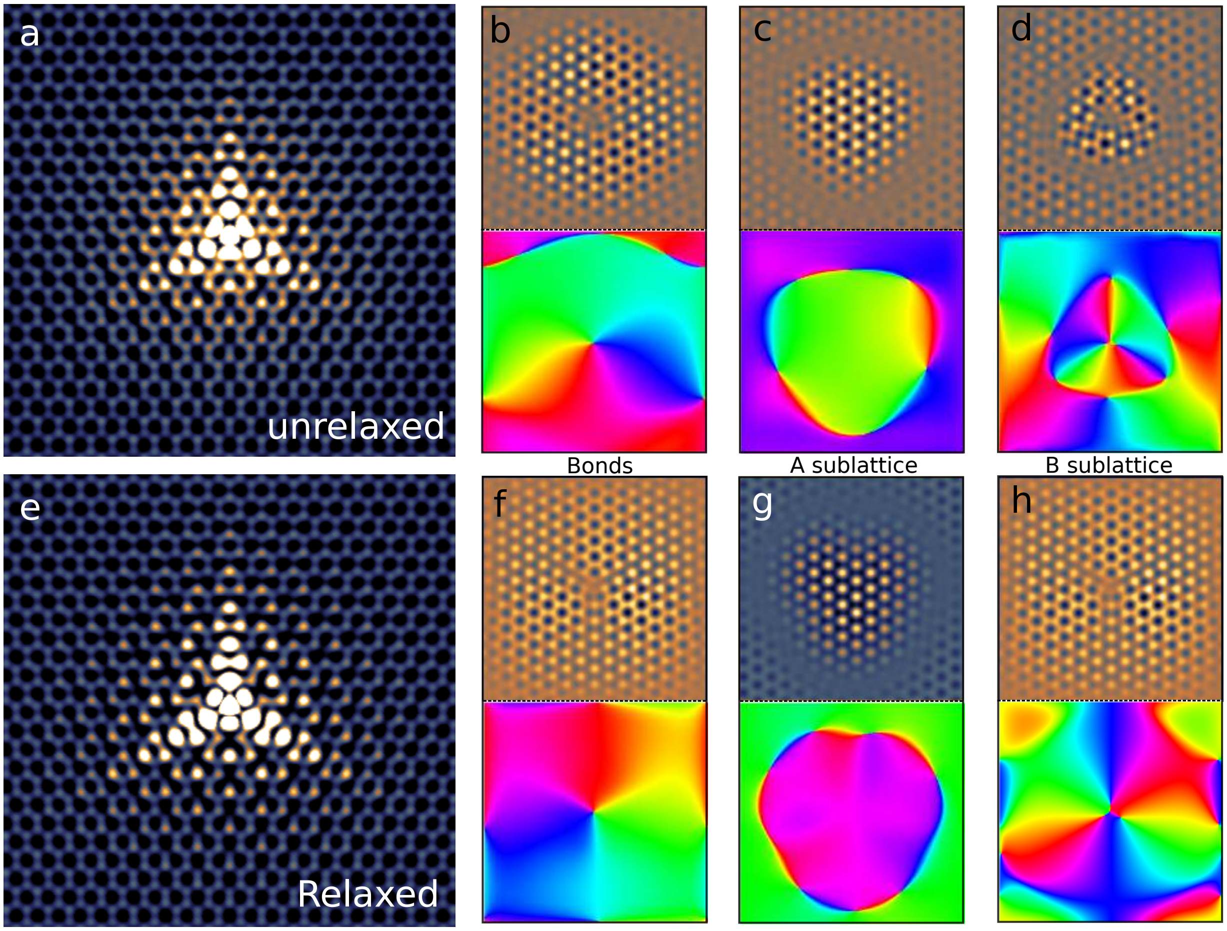

Supplementary Figure 6: The effect of structural relaxation near an H atom from DFT calculations. a, STM image of H adatom in graphene without structural relaxation simulated using DFT. The signal is decomposed into the contributions of the bonds (**b**), A sublattice (**c**) and B sublattice (**d**). In each case we show the signal and the corresponding phase as extracted by the method described in Supplementary Note 5. **e,** Same image with structural relaxation. The structural relaxation does not affect the Kekulé vortex. The signal is decomposed into the contributions of the bonds (**f**), A sublattice (**g**) and B sublattice (**h**).

Supplementary Note 1 : Definition of the bond order operator

To describe the Kekulé order and its local variations one needs not only the on-site contributions to LDOS, but also the information about the phase difference between wavefunction amplitudes on the nearest-neighbor sites. To achieve this, we define a bond-order operator in the atomic orbital basis by taking the orbital wave function as an envelope $f(\mathbf{r})$. The LDOS of electrons at position $\mathbf{r}$ between atoms i, j is written:

$$\begin{aligned} \rho_{i,j}(\mathbf{r}) &= |\phi_i(\mathbf{r}) + \phi_j(\mathbf{r})|^2 \\ &= |\psi_i f(\mathbf{r} - \mathbf{r_i}) + \psi_j f(\mathbf{r} - \mathbf{r_j})|^2, \end{aligned} \tag{1}$$

where ψ_i is the wavefunction projected to the orbital basis $\phi(r) = \sum_i \psi_i f(\mathbf{r} - \mathbf{r}_j)$. As an approximation, we use the LDOS at the middle of the bond to represent the bond ordering:

$$\begin{aligned} \rho_{ij} &= |\psi_i f(a_0/2) + \psi_j(\mathbf{r}) f(a_0/2)|^2 \\ &= (|\psi_i|^2 + |\psi_j|^2 + Re\langle\psi_i|\psi_j\rangle) f^2(a_0/2), \end{aligned} \tag{2}$$

where the overlap term $b_{ij} = Re\langle\psi_i|\psi_j\rangle$ is used to depict the bond ordering. The winding of the bond order originates from the intervalley scattering in the continuum model of graphene. Typically, since the bonds are present between the A and B atoms, the modulation of bond order is affected by the inter-sublattice Green's function component and b_{ij} can be as written as

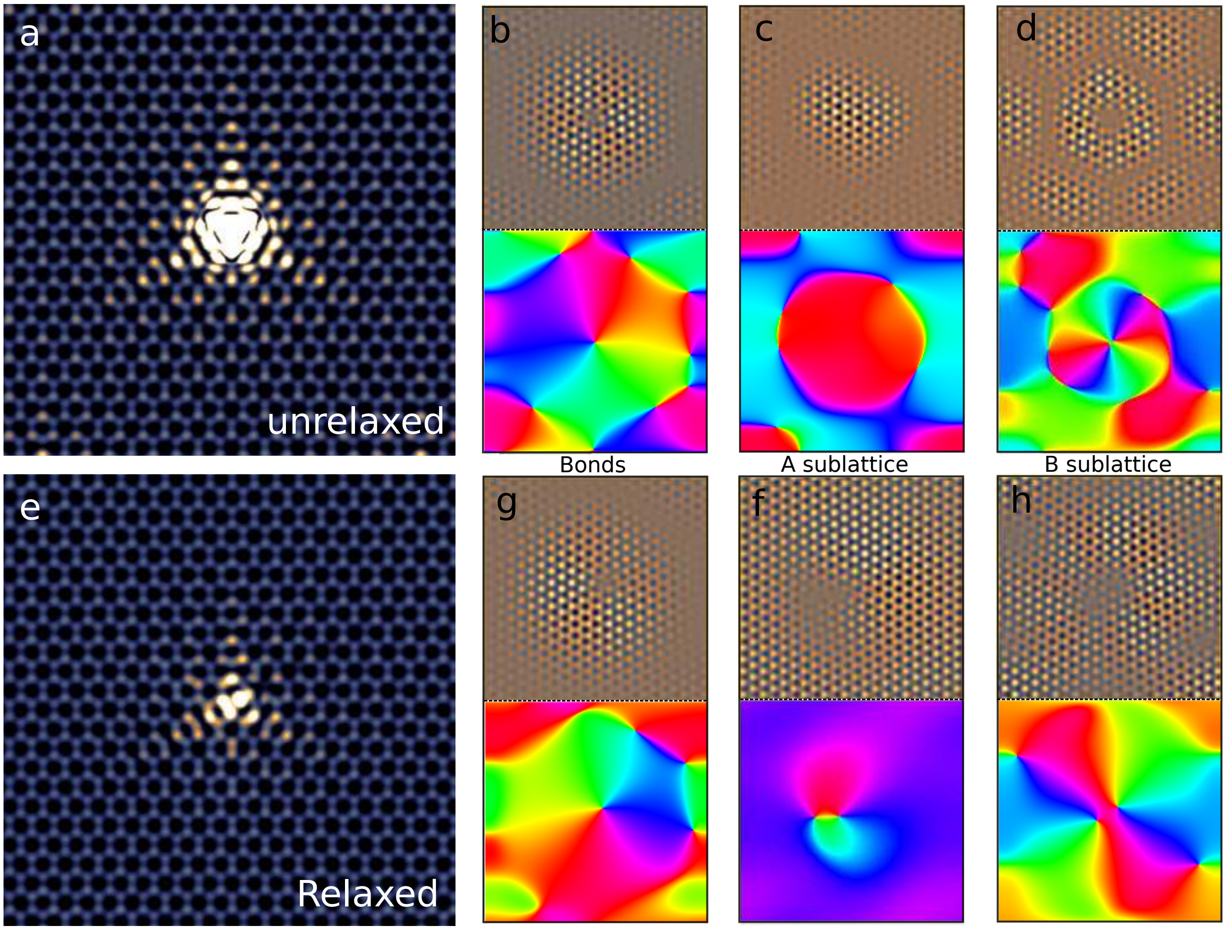

Supplementary Figure 7: **DFT calculations of the local density of states at a vacancy defect in graphene. a,** STM image of a vacancy defect in graphene without structural relaxation simulated by DFT. The signal is decomposed into the contributions of the bonds (**b**), A sublattice (**c**) and B sublattice (**d**). In each case we show the signal and the corresponding phase as extracted by the method described in section . **e,** Same image, but with structural relaxation. The signal is decomposed into the contributions of the bonds (**f**),A sublattice (**g**) and B sublattice (**h**). Both show a Kekulé vortex even if the structural relaxation breaks C_3 symmetry.

$$b_{ij} = \sum_n \langle \psi_i^n | \psi_j^n \rangle + h.c. = Re\{G_{ij}\},$$

We present the detailed calculation of this term in Supplementary Note 2.

Supplementary Note 2 : LDOS modulations from low-energy Green's function T-matrix approach

We describe the p_z states of graphene through the nearest-neighbor tight-binding (TB) Hamiltonian

$$H = \sum_{\langle i,j \rangle} t_{ij} c_i^\dagger c_j + h.c., \tag{3}$$

where the $c_i^{(\dagger)}$ operator annihilates (creates) a p_z electron on site i. We model the H adatom by the onsite potential $V = V_0 c_0^\dagger c_0$. Here we define the adatom site as lattice origin and assume it lies on sublattice A. We consider the Bloch Hamiltonian in momentum space

$$H(\boldsymbol{k}) = \begin{pmatrix} 0 & h(\boldsymbol{k}) \\ h^\dagger(\boldsymbol{k}) & 0 \end{pmatrix}, \tag{4}$$

with $h(\boldsymbol{k}) = t[\exp(i\boldsymbol{k}\cdot\boldsymbol{d}_1) + \exp(i\boldsymbol{k}\cdot\boldsymbol{d}_2) + \exp(i\boldsymbol{k}\cdot\boldsymbol{d}_3)]$, where $\boldsymbol{d}_1 = (\sqrt{3}, 1)/2$, $\boldsymbol{d}_2 = (-\sqrt{3}, 1)/2$, and $\boldsymbol{d}_3 = (0, -1)$ are the three nearest-neighbor vectors given in units of the lattice constant.

We then describe the elastic scattering of electrons using a T-matrix approach based on Green's functions. We introduce the bare retarded Green's function in momentum space as $G(\boldsymbol{k}, \omega) = [\omega - H(\boldsymbol{k})]^{-1}$. Considering only the massless relativistic electrons in valley $\boldsymbol{K}^{\xi}_{mn}$ leads to the real-space representation[1]

$$G(\boldsymbol{K}^{\xi}_{mn}, \boldsymbol{r}, \omega) \simeq -\frac{\omega e^{i\boldsymbol{K}^{\xi}_{mn}\cdot\boldsymbol{r}}}{(2v_{\rm F}a_0)^2} \begin{pmatrix} iH_0(q_{\rm F}r) & \xi H_1(q_{\rm F}r)\, e^{i\boldsymbol{K}^{\xi}_{mn}\cdot\boldsymbol{d}_3 + i\xi\theta_{\boldsymbol{r}}} \\ \xi H_1(q_{\rm F}r)\, e^{-i\boldsymbol{K}^{\xi}_{mn}\cdot\boldsymbol{d}_3 - i\xi\theta_{\boldsymbol{r}}} & iH_0(q_{\rm F}r) \end{pmatrix}, \quad (5)$$

where $\boldsymbol{K}^{\xi}_{mn} = \xi(\boldsymbol{b}_1 - \boldsymbol{b}_2)/3 + m\boldsymbol{b}_1 + n\boldsymbol{b}_2$, $\xi = \pm 1$ is the valley index, and $\boldsymbol{b}_{1,2}$ are basis vectors of the reciprocal Bravais lattice (see Supplementary Figure 8). The wavevector $\boldsymbol{q}_{\rm F}$ relates to energy ω through the Dirac dispersion relation $\omega = v_{\rm F}a_0q_{\rm F}$. The representation basis refers to the p_z orbitals of carbon atoms on the two sublattices. We consider the quasiparticle interference in the middle of covalent bonds. There are four nonequivalent contributions

$$\begin{aligned}
\Delta G_{AA} &= G_{AA}(\boldsymbol{K}^{\xi}_{mn}, \boldsymbol{r}, \omega)T_A(\omega)G_{AA}(\boldsymbol{K}^{\xi'}_{m'n'}, -\boldsymbol{r}, \omega) = -\frac{\omega^2}{(2v_{\rm F}a_0)^4}T_A(\omega)H_0(q_{\rm F}r)H_0(q_{\rm F}r)\, e^{i\boldsymbol{\Delta K}\cdot\boldsymbol{r}}, \quad (6)\\
\Delta G_{BB} &= G_{BA}(\boldsymbol{K}^{\xi}_{mn}, \boldsymbol{r}, \omega)T_A(\omega)G_{AB}(\boldsymbol{K}^{\xi'}_{m'n'}, -\boldsymbol{r}, \omega) = -\frac{\omega^2}{(2v_{\rm F}a_0)^4}T_A(\omega)H_1(q_{\rm F}r)H_1(q_{\rm F}r)\, e^{i\boldsymbol{\Delta K}\cdot\boldsymbol{r} - i\boldsymbol{\Delta K}\cdot\boldsymbol{d}_3 - i\Delta\xi\theta_{\boldsymbol{r}}}\ (\xi\xi'),\\
\Delta G_{AB} &= G_{AA}(\boldsymbol{K}^{\xi}_{mn}, \boldsymbol{r}, \omega)T_A(\omega)G_{AB}(\boldsymbol{K}^{\xi'}_{m'n'}, -\boldsymbol{r}, \omega) = -\frac{\omega^2}{(2v_{\rm F}a_0)^4}T_A(\omega)H_0(q_{\rm F}r)H_1(q_{\rm F}r)\, e^{i\boldsymbol{\Delta K}\cdot\boldsymbol{r} + i\boldsymbol{K}^{\xi'}_{m'n'}\cdot\boldsymbol{d}_3 + i\xi'\theta_{\boldsymbol{r}}}\ (i\xi'),\\
\Delta G_{BA} &= G_{BA}(\boldsymbol{K}^{\xi}_{mn}, \boldsymbol{r}, \omega)T_A(\omega)G_{AA}(\boldsymbol{K}^{\xi'}_{m'n'}, -\boldsymbol{r}, \omega) = +\frac{\omega^2}{(2v_{\rm F}a_0)^4}T_A(\omega)H_0(q_{\rm F}r)H_1(q_{\rm F}r)\, e^{i\boldsymbol{\Delta K}\cdot\boldsymbol{r} - i\boldsymbol{K}^{\xi}_{mn}\cdot\boldsymbol{d}_3 - i\xi\theta_{\boldsymbol{r}}}\ (i\xi)\ .
\end{aligned}$$

The two first equations correspond to the usual backscattering processes, while the two last equations describe loop-scattering processes allowed by the overlap of the p_z orbitals on the bonds. We now focus on elastic scattering between the nearest-neighbor valleys. There are three scattering wavevectors $\boldsymbol{\Delta K}_1$, $\boldsymbol{\Delta K}_2$ and $\boldsymbol{\Delta K}_3$ (see Supplementary Figure 8), as well as their three conjugates obtained through time-reversal symmetry. The standing-wave pattern in the LDOS resulting from the nearest-neighbor intervalley scattering is obtained as $\Delta\rho = -{\rm Im}[\Delta G]/\pi$ and verifies

$$\begin{aligned}
\Delta\rho_{AA}(\boldsymbol{r}, \omega) &= A(r, \omega)\,[\cos(\boldsymbol{\Delta K}_1\cdot\boldsymbol{r}) + \cos(\boldsymbol{\Delta K}_2\cdot\boldsymbol{r}) + \cos(\boldsymbol{\Delta K}_3\cdot\boldsymbol{r})], \quad (7)\\
\Delta\rho_{BB}(\boldsymbol{r}, \omega) &= B(r, \omega)\,[\cos(\boldsymbol{\Delta K}_1\cdot\boldsymbol{r} - 2\theta_{\boldsymbol{r}} + \pi) + \cos(\boldsymbol{\Delta K}_2\cdot\boldsymbol{r} - 2\theta_{\boldsymbol{r}} + \pi/3) + \cos(\boldsymbol{\Delta K}_3\cdot\boldsymbol{r} - 2\theta_{\boldsymbol{r}} - \pi/3)],\\
\Delta\rho_{AB}(\boldsymbol{r}, \omega) + \Delta\rho_{BA}(\boldsymbol{r}, \omega) &= C(r, \omega)\,[\sin(\boldsymbol{\Delta K}_1\cdot\boldsymbol{r} - \theta_{\boldsymbol{r}} + \pi) + \sin(\boldsymbol{\Delta K}_2\cdot\boldsymbol{r} - \theta_{\boldsymbol{r}} - \pi/3) + \sin(\boldsymbol{\Delta K}_3\cdot\boldsymbol{r} - \theta_{\boldsymbol{r}} + \pi/3)]\ ,
\end{aligned}$$

where we introduce the isotropic energy-dependent pre-factors

$$\begin{aligned}
A(r, \omega) &= \frac{\omega^2}{\pi(2v_{\rm F}a_0)^4}{\rm Im}\,[T_A(\omega)H_0(q_{\rm F}r)H_0(q_{\rm F}r)]\,,\\
B(r, \omega) &= \frac{\omega^2}{\pi(2v_{\rm F}a_0)^4}{\rm Im}\,[T_A(\omega)H_1(q_{\rm F}r)H_1(q_{\rm F}r)]\,,\\
C(r, \omega) &= \frac{\omega^2}{\pi(2v_{\rm F}a_0)^4}{\rm Im}\,[T_A(\omega)H_0(q_{\rm F}r)H_1(q_{\rm F}r)]\,.
\end{aligned}$$

This low-energy analytical description shows good agreement with the DFT simulations presented in the main text, as well as the tight-binding calculations in Supplementary Figure 4.

Supplementary Note 3 : Relationship between the Kekulé order and Clar's sextet configurations

Following the definition of bond order (Supplmentary Equation 2), the Kekulé ordering can be defined through an intervalley coherence

$$|Kek(\phi)\rangle = |KA\rangle + e^{i\phi}|K'B\rangle, \quad (8)$$

where angle ϕ identifies one of the three Kekulé domains. Likewise, Supplementary Equation 8 can also be related to the Clar's sextet description. As shown in Supplementary Figure 9, the three distinct configurations of Clar's sextets are generated by angles $\phi = \pi, \pi/3, -\pi/3$. Such a correspondence between the intervalley phase and Kekulé domains gives explicitly the variation of local Kekulé orders emerging from the intervalley scattering (Supplementary Equation 7).

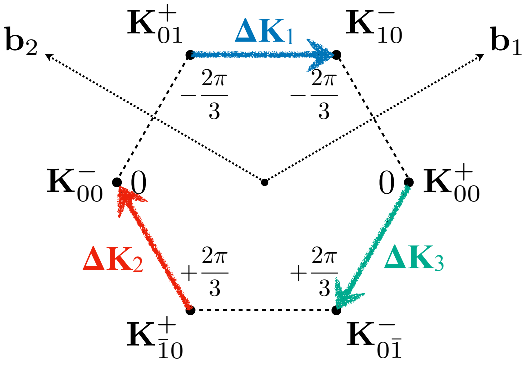

Supplementary Figure 8: **Scattering pathways in graphene.** The dashed hexagon outlines the Brillouin zone with valleys $\boldsymbol{K}^{\xi}_{mn}$ in the corners. The dotted arrows represent the basis vectors $\boldsymbol{b}_1$ and $\boldsymbol{b}_2$ that span the reciprocal lattice. The colored arrows show the three scattering wavevectors $\boldsymbol{\Delta K}_1$, $\boldsymbol{\Delta K}_2$ and $\boldsymbol{\Delta K}_3$. The numbers inside the hexagon correspond to the valley phase shifts $\boldsymbol{K}^{\xi}_{mn} \cdot \boldsymbol{d}_3$.

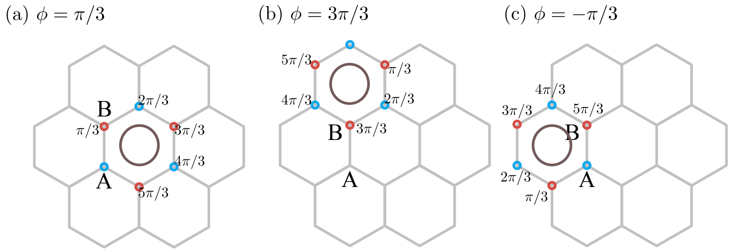

Supplementary Figure 9: Three equivalent Clar's sextet configurations resulting from intervalley coherence. The angle ϕ identifies with one of the three Kekulé domains.

Supplementary Note 4 : Robustness of fractionalization

The vortex-like order parameter predicts a fractional excess charge $e/2$ of spinless electrons in the limit of exact particle-hole (PH) symmetry. In realistic graphene samples, the symmetry is not strictly preserved. Potential particle-hole breaking may come from different origins. It is known that the finite second nearest neighbor hoppings in graphene will break the PH symmetry. Also, while we model the adatom by forbidding the hopping to the p_z orbital, some real defects may not be exactly PH-symmetric. As a consequence, a hydrogen adatom will likely induce an irrational excess charge, which is regarded as a two-dimensional soliton mode.

We discuss the possibility that bond distortion originating from the Kekulé texture through a Fock-like term[2]

$$H' = -V \sum_{\langle i,j \rangle} c_i^\dagger c_j c_j^\dagger c_i. \tag{9}$$

Within the mean-field approximation H'writes

$$H'_{mf} \propto -V' \langle c_i^\dagger c_j \rangle, \tag{10}$$

which is exactly in the form of intervalley-scattering corrections to the bond order. Such a mechanism (Supplementary Equation 10) suggests a potential emergent Kekulé pattern in the hopping integrals as a consequence of the bond order modulation.

We finally provide our evaluation of particle-hole asymmetry from the next-nearest neighbor hoppings t'. In the Slater-Koster model of graphene, the 2nd order hopping is approximately $t' = 0.1t$.[3] Hopping t' is to be compared with the energy scale of the zero mode. The factor Δ_0 is brought as the vortex amplitude: $\Delta(r) = \Delta_0(r)e^{ni\theta}$. We approximate its value as 100 meV, or $t/30$. Therefore, the final estimation is

$$\delta_\varepsilon = \frac{t'\Delta_0}{t^2}\Delta_0 \approx \frac{1}{300}\Delta_0 = 0.3\ \text{meV}$$

The influence on charge fractionalization is then $\Delta_e = \mathrm{DOS}(E=0) \times \delta_\varepsilon$. Since the broadening is of the order of 10 meV, such δ_ε would not change the $e/2$ charge drastically. The conclusion is also consistent with the previous results in Ref.[4].

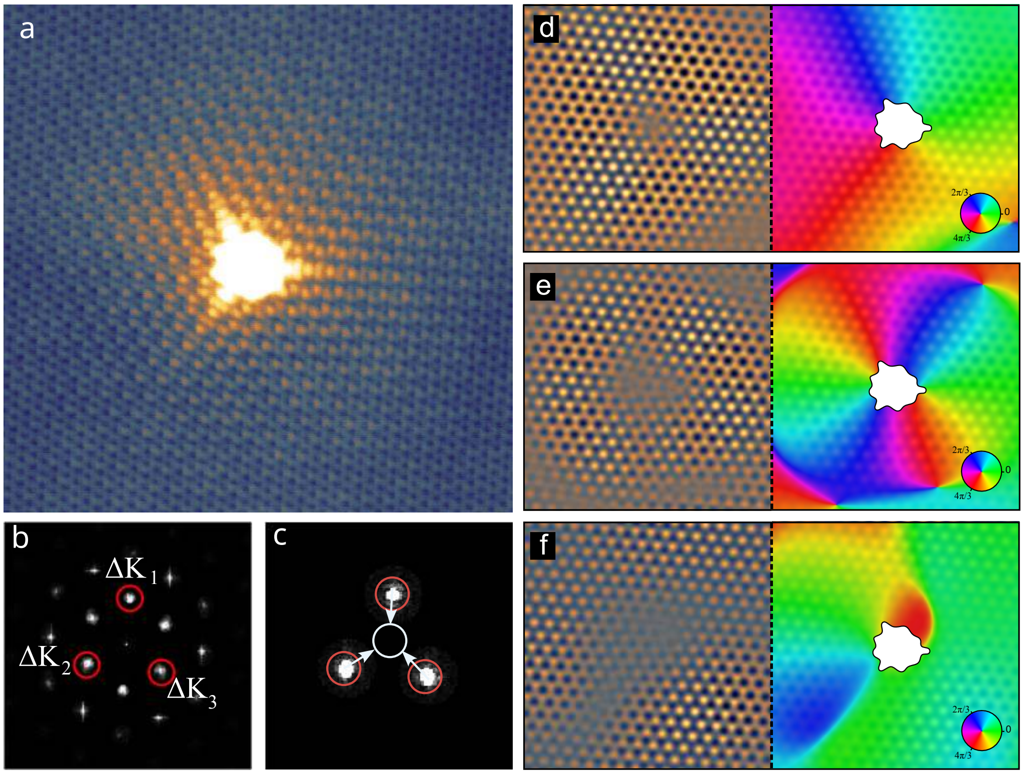

Supplementary Figure 10: **Extraction of the Kekulé order parameter of a H adatom on graphene. a,** An STM image of a H adatom on graphene (identical to Fig. 1b of the main text). **b** Fourier transform of the STM image, with $\boldsymbol{\Delta K}_{n=1,2,3}$ being the three intervalley scattering peaks related by C_3 rotation. **c**. In our GPA analysis, the intervalley peaks are shifted to the Γ point to extract the low-frequency signals. **d** Extracted signal on the bond-centered contribution and its phase **e**. Extracted signal on the B sublattice and its phase. **f**. Extracted signal on the A sublattice and its phase.

Supplementary Note 5 : Extraction of the Kekulé order parameter with GPA

The quantitative analysis of the order parameters was performed using a modified version of the geometric phase analysis (GPA) introduced by Hytch *et al.*[5]. The aim is to determine the phase of an oscillating signal in real space by means of a Fourier-space filtering. Using a filter centered on some harmonic $\boldsymbol{k}_0$ in momentum space leads to a complex signal in real space through inverse Fourier transform. The argument of this complex signal then corresponds to the local phase of the initial oscillating signal with harmonic $\boldsymbol{k}_0$. Note that the plane-wave contribution $\boldsymbol{k}_0 \cdot \boldsymbol{r}$ is a part of the extracted phase. This can obscure slow variations of the phase. Thus, we subtract $\boldsymbol{k}_0 \cdot \boldsymbol{r}$ to the phase and obtain the so-called reduced phase in the GPA language. Alternatively, this contribution can be removed by shifting the Fourier signal centered at $\boldsymbol{k}_0$ to the $\boldsymbol{\Gamma}$ point to perform inverse Fourier transform.

In our system, the Kekulé modulation is evidenced in Fourier space. It corresponds to the existence of the high-intensity peaks at the six intervalley scattering wave-vectors $\pm\boldsymbol{\Delta K}_1$, $\pm\boldsymbol{\Delta K}_2$, and $\pm\boldsymbol{\Delta K}_3$ (see Supplementary Figure 8). The time conjugation relate $\boldsymbol{\Delta K}_n$ to $-\boldsymbol{\Delta K}_n$, thus we focus only on the three scattering wavevectors $\boldsymbol{\Delta K}_1$, $\boldsymbol{\Delta K}_2$, and $\boldsymbol{\Delta K}_3$. It is then tempting to apply the GPA directly at these points to extract the local phase and so reveal whether a vortex is associated with the Kekulé modulation. However, a complication arises in our system. The signal to be filtered around some $\boldsymbol{\Delta K}_n$ consists of the superposition of three distinct contributions — from sublattice A, sublattice B, and the bond-centered contribution. Here, we would like to separate each of them to access only the bond-centered contribution.

C_3	$\boldsymbol{\Delta K}_1$	$\boldsymbol{\Delta K}_2$	$\boldsymbol{\Delta K}_3$
$\Delta\rho_A$	1	1	1
$\Delta\rho_B$	1	ω^2	ω
$\Delta\rho_{AB}$	1	ω	ω^2
$\Delta\rho_{BA}$	1	ω	ω^2

Supplementary Table I: Effect of C3 symmetry on each LDOS contributions in the vicinity of the intervalley scattering wavevectors $\boldsymbol{\Delta K_1}$, $\boldsymbol{\Delta K_2}$, and $\boldsymbol{\Delta K_3}$. The columns shows how each LDOS contribution in $\boldsymbol{\Delta K_2}$ and $\boldsymbol{\Delta K_3}$ relate to that in $\boldsymbol{\Delta K_1}$, which we take as reference and fixe to 1. Thus, each contribution transforms differently from the others under C3 symmetry by picking a global phase 1, ω, or ω^2, where $\omega = \exp(i2\pi/3)$.

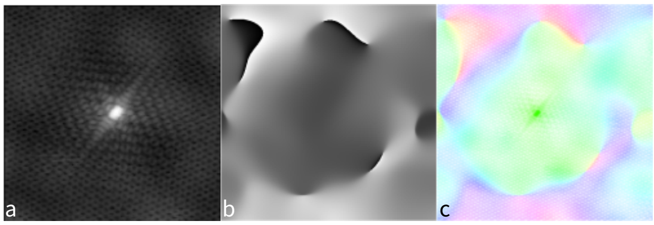

Supplementary Figure 11: **Order parameter extracted from the STM image of a divacancy defect in graphene. a,** Greyscale STM image a divacancy defect in graphene. **b,** The phase of bond order parameters **c,** The bond parameter overlayed on the STM image.

To extract the bond-centered contribution from the STM signal at intervalley scattering wavevectors, we exploit the system symmetry. The H adatom chemisorbed on a site in sublattice A preserves an important symmetry of graphene, C_3. The threefold invariance also appears in the Fourier transform of the electronic signal $\Delta\rho(\boldsymbol{k})$. In particular, the C_3 operator relates the peaks at the three intervalley scattering wavevectors $\boldsymbol{\Delta K}_1$, $\boldsymbol{\Delta K}_2$, and $\boldsymbol{\Delta K}_3$ (see Supplementary Table I). We write the total signal as the sum of sublattice A, sublattice B, and bond contributions: $\Delta\rho = \Delta\rho_A + \Delta\rho_B + \Delta\rho_{\rm bond}$ with $\Delta\rho_{\rm bond} = \Delta\rho_{AB} + \Delta\rho_{BA}$. For instance. from Supplementary Equations 6, we find that the contributions transform under C_3 in the following way where $\omega = \exp i2\pi/3$. Thus, the contributions $\Delta\rho_A$, $\Delta\rho_B$, and $\Delta\rho_{\rm bond}$ each transform under different irreducible representations of the C$_3$ point group. This key argument now allows us to resolve each contribution individually in Fourier space, by analogy to methods introduced in Refs. 6,7. Indeed, it is now relevant to expand the total signal in the vicinity of the three intervalley scattering wavevectors as

$$\big(\Delta\rho[\boldsymbol{\Delta K}_1], \Delta\rho[\boldsymbol{\Delta K}_2], \Delta\rho[\boldsymbol{\Delta K}_3]\big) = \Delta\rho_A\ (1,1,1) + \Delta\rho_B\ (1,\omega^2,\omega) + \Delta\rho_{\rm bond}\ (1,\omega,\omega^2)\ , \tag{11}$$

where the order parameter $\Delta\rho_{\rm bond}$ is a complex scalar field that characterizes the Kekulé bond modulations. Thus, we obtain the bond-order parameter from the STM as a linear combination of the three high-intensity peaks

$$\Delta\rho_{\rm bond} = \Delta\rho[\boldsymbol{\Delta K}_1] + \omega^2\ \Delta\rho[\boldsymbol{\Delta K}_2] + \omega\ \Delta\rho[\boldsymbol{\Delta K}_3]\ . \tag{12}$$

Performing the inverse Fourier transform leads to the real-space representation of the bond-order parameter in Supplementary Figure 10e. The argument reveals that the phase of the Kekulé order parameter winds 2π around the H adatom. This demonstrates the existence of the Kekulé vortex. In the same vein, we can also extract from the STM data the onsite contributions of sublattices A and B. We recover that the intervalley modulation is trivial on sublattice A (Supplementary Figure 10d) and exhibit a 4π vortex on sublattice B (Supplementary Figure 10f), which is consistent with previous works[1,8].

Finally, we present the order parameters of a divacancy (Supplementary Figure 11), a single vacancy (Supplementary Figure 7) and a H adatom (Supplementary Figure 6). Supplementary Figure 11 demonstrates that the divacancy defect does not induce a winding of the Kekulé bond ordering. The DFT-simulated result in Supplementary Figure 7 further suggests that the vacancy defect in graphene will induce the same order parameter winding as the hydrogen adatom: a 2π vortex in the bond order and 4π vortex in the on-site order parameter. The Kekulé vortex is present even in the case of the reconstructed vacancy defect that breaks the C_3 symmetry and breaks the hole-particle symmetry to a significant extent.

Supplementary References

---

* Electronic address: ivan.brihuega@uam.es
† Electronic address: oleg.yazyev@epfl.ch
‡ Electronic address: vincent.renard@cea.fr

[1] Dutreix, C. & Katsnelson, M. I. Friedel oscillations at the surfaces of rhombohedral N-layer graphene. Phys. Rev. B **93**, 035413 (2016). URL `https://link.aps.org/doi/10.1103/PhysRevB.93.035413`.

[2] Hou, C.-Y., Chamon, C. & Mudry, C. Electron Fractionalization in Two-Dimensional Graphenelike Structures. Phys. Rev. Lett. **98**, 186809 (2007).

[3] Kretinin, A. et al. Quantum capacitance measurements of electron-hole asymmetry and next-nearest-neighbor hopping in graphene. Phys. Rev. B **88**, 165427 (2013). URL `https://link.aps.org/doi/10.1103/PhysRevB.88.165427`.

[4] Brihuega, I. et al. Quasiparticle Chirality in Epitaxial Graphene Probed at the Nanometer Scale. Phys. Rev. Lett. **101**, 206802 (2008).

[5] Hÿtch, M., Snoeck, E. & Kilaas, R. Quantitative measurement of displacement and strain fields from HREM micrographs. Ultramicroscopy **74**, 131–146 (1998). URL `https://www.sciencedirect.com/science/article/pii/S0304399198000357`.

[6] Nuckolls, K. P. et al. Quantum textures of the many-body wavefunctions in magic-angle graphene. Nature **620**, 525–532 (2023). URL `http://dx.doi.org/10.1038/s41586-023-06226-x`.

[7] Călugăru, D. et al. Spectroscopy of Twisted Bilayer Graphene Correlated Insulators. Phys. Rev. Lett. **129**, 117602 (2022). URL `https://link.aps.org/doi/10.1103/PhysRevLett.129.117602`.

[8] Dutreix, C. & *et al.* Measuring the Berry phase of graphene from wavefront dislocations in Friedel oscillations. Nature **574**, 219–222 (2019).
